# Survival and mortality among users and non-users of hydroxyurea with sickle cell disease

**DOI:** 10.1590/0104-1169.3385.2526

**Published:** 2015

**Authors:** Olinda Maria Rodrigues de Araujo, Maria Lúcia Ivo, Marcos Antonio Ferreira, Elenir Rose Jardim Cury Pontes, Ieda Maria Gonçalves Pacce Bispo, Eveny Cristine Luna de Oliveira

**Affiliations:** 1 PhD, Adjunct Professor, Universidade Federal de Mato Grosso do Sul, Campo Grande, MS, Brazil; 2 PhD, Associate Professor, Universidade Federal de Mato Grosso do Sul, Campo Grande, MS, Brazil; 3 PhD, Adjunct Professor, Universidade Federal do Rio Grande do Norte, Natal, RN, Brazil; 4 MSc, RN, Universidade Federal de Mato Grosso do Sul, Três Lagoas, MS, Brazil; 5 Doctoral student, Universidade Federal de Mato Grosso do Sul, Campo Grande, MS, Brazil. Physician, Universidade Federal de Mato Grosso do Sul, Campo Grande, MS, Brazil

**Keywords:** Survivorship (Public Health), Mortality, Hemoglobin, Sickle, Hydroxyurea, Nursing

## Abstract

**OBJECTIVE:**

to estimate survival, mortality and cause of death among users or not of hydroxyurea with sickle cell disease.

**METHOD:**

cohort study with retrospective data collection, from 1980 to 2010 of patients receiving inpatient treatment in two Brazilian public hospitals. The survival probability was determined using the Kaplan-Meier estimator, survival calculations (SPSS version 10.0), comparison between survival curves, using the log rank method. The level of significance was p=0.05.

**RESULTS:**

of 63 patients, 87% had sickle cell anemia, with 39 using hydroxyurea, with a mean time of use of the drug of 20.0±10.0 years and a mean dose of 17.37±5.4 to 20.94±7.2 mg/kg/day, raising the fetal hemoglobin. In the comparison between those using hydroxyurea and those not, the survival curve was greater among the users (p=0.014). A total of 10 deaths occurred, with a mean age of 28.1 years old, and with Acute Respiratory Failure as the main cause.

**CONCLUSION:**

the survival curve is greater among the users of hydroxyurea. The results indicate the importance of the nurse incorporating therapeutic advances of hydroxyurea in her care actions.

## Introduction

Sickle cell disease (SCD) is a generic term attributed to a group of hereditary diseases with a predominance of hemoglobin S, and is among the most frequently-found genetic diseases in the human population^(^ 1 ^)^. The clinical presentation is characterized by two key physiopathological processes of sickle cell anemia: hemolysis and vaso-occlusion^(^ 2 ^)^. These processes occur from the first year of life onward and, over the years, due to the chronic nature of the disease, the severity worsens, injuring various tissues and organs^(^ 3 ^)^.

Currently, advances in the treatment of, and studies on the survival of, sickle cell patients demonstrate that life expectancy has considerably improved^(^ 4 ^)^_._ Among the therapeutic options available, besides bone marrow transplant and chronic transfusion, one can highlight hydroxyurea (HU)^(^ 5 ^)^, whose action can increase the levels of fetal hemoglobin, improving the clinical severity and the hematological parameters, as well as reducing the rates of the disease's morbidity and mortality, with an increase in survival^(^ 6 ^-^ 7 ^)^.

In this regard, emphasis is placed on a study undertaken in the United States and in Canada with patients participating in the MSH study (The Multicenter Study of Hydroxyurea in Sickle Cell Anemia), which made it possible to analyze the impact of the use of HU on mortality, with a 40% reduction in mortality being recorded by the researchers (p=0.04) among the users of this medication over nine years of monitoring^(^ 8 ^)^. 

In the light of the seriousness of SCD, and bearing in mind the lack of nursing publications on this issue^(^ 3 ^-^ 9 ^)^, in particular with HU, it falls to the nurse to be familiar with the advances of this therapy, which have contributed to the reduction of mortality and to the consequent increase in survival in this clientele. Published evidence has demonstrated that the principal therapeutic approach in sickle cell anemia is to try to alter the production of hemoglobin S to fetal hemoglobin. This results in a lesser degree of severe hemolytic anemia, and fewer symptoms^(^ 11 ^)^.

This study's contribution is to bring support to the nurse for her work in health surveillance for the patient with sickle cell disease, ranging from guidance on medication through to the monitoring of the strategy of self-administration of HU by the patient. Thus, this study's objective was to calculate the survival, mortality and cause of death among users or not of hydroxyurea with sickle cell disease. 

## Method

This is a cohort study with retrospective data collection, involving patients diagnosed with sickle cell disease attended in two public hospitals in the Brazilian state of Mato Grosso do Sul in the period 1980 - 2010. 

Data collection was undertaken in the Medical Records Service (SAME) of the above-mentioned hospitals in November 2010 - October 2011, through consulting the medical records of patients with hemoglobinopathies who were attended in the Hematology Services. A total of 63 patients was included, of all ages, with a medical diagnosis of sickle cell disease confirmed by hemoglobin electrophoresis, who met the inclusion criteria. Those who presented other hemoglobinopathies and sickle cell trait were excluded. 

A data collection was undertaken by one of the study's researchers through the use of an instrument containing the following variables: characterization of the sample (medical diagnosis, sex and age); survival (date of diagnosis, follow-up period following admission to the service, outcomes - deaths and patient's discontinuation of treatment); use or not of hydroxyurea (initial and final doses, age at the time of indication of the medication, levels of fetal hemoglobin prior to and after the use of HU); mortality (sex, age, genotype, number of deaths and their causes).

The data were organized in an Excel(r) spreadsheet, and the descriptive measures were calculated with the use of the SAS (Statistical Analysis System) program for Windows, version 9.0. In order to determine the probability of survival, the Kaplan-Meier method was used, the date of the confirmation of the medical diagnosis considered as the initial point and death or discontinuation of treatment as the closing point. The survival calculations were undertaken using the SPSS software (Statistical Package for the Social Sciences) version 10.0 and, for comparison of the survival curves, the log rank method was used. The level of significance considered for the study was 0.05. The Mann-Whitney test was used for comparing the length of use of HU between the sexes. 

 The study was approved regarding its ethical and methodological aspects by the Research Ethics Committee of the Federal University of Mato Grosso do Sul, under protocol N. 1.822/2010.

## Results

The 63 patients with SCD included in this cohort, were monitored over 30 years, from 1980 to 2010. Of these, 55 (87.3%) had sickle cell anemia, followed by eight with Sickle Cell hemoglobinopathy, which are compound heterozygotes, of whom 38 (60.3%) were female and 25 (39.7%) male, aged between five and 63 years old. It is emphasized that the eight cases of SC hemoglobinopathy found in the period researched were included in the study as it dealt with survival in SCD, in the group of non-users of hydroxyurea. Of the 63 patients, 39 used HU, with the mean exposure being six years. At the time the medication was indicated, the mean age was 20.0±10.0 years. The mean initial dosage of HU was 17.37±5.4mg/kg/day; at the end of the period investigated, it was 20.94±7.2mg/kg/day.

In relation to fetal hemoglobin prior to the use of HU, a mean was obtained of 7.73±5.1 and, following use, there was a significant increase to 14.31±7.4, p<0.001.

**Figure 1 -:**
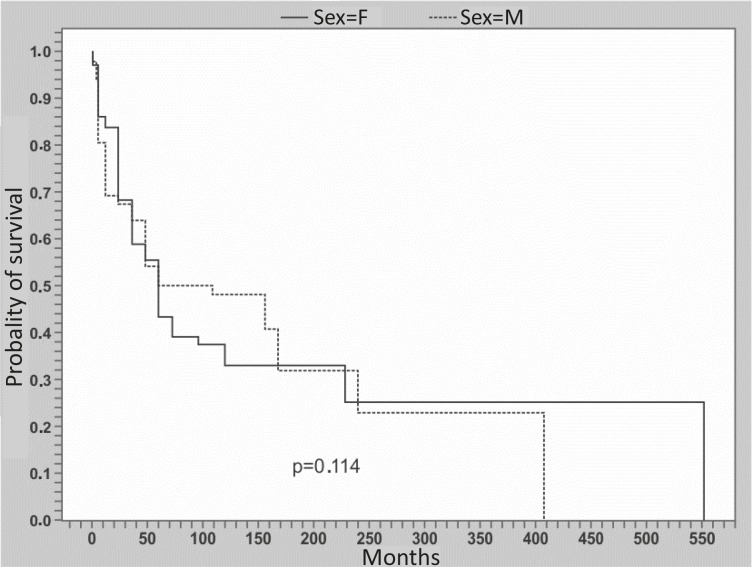
Estimated probability curve for survival, by sex, in two public hospitals in the state of Mato Grosso do Sul, Brazil, between 1980 and 2010 (n=63)

The accumulated probability of survival was calculated based on the total of patients studied (n=63), among whom 48 were being monitored in the service (76.2%), 10 died (15.8%) and five discontinued treatment in the institution (8%). Time zero (initial) was considered to be the moment of diagnosis, while the closure was the patient's death or discontinuation of treatment. Table 1 describes the data found for global survival, organized by sex, and establishes whether there was or was not difference between the two groups. At 24 months (two years), the accumulated probability of survival was 74%; at 48 months (four years), it was 61%; at 120 months (10 years), it was 42%; at 240 months (20 years), it was 31%; and at 480 months (40 years), it was 25%. There was no statistically significant difference between men and women (log rank =0.114). In establishing global survival by sex, one can observe a slightly greater survival curve in the women in the first two years, which following that is inverted until closure (Figure 1).

**Table 1 -:** Accumulated probability of global survival of patients with sickle cell disease, by sex, in two public hospitals in the state of Mato Grosso do Sul, Brazil, between 1980 and 2010 (N= 63)

Follow-up time (months)	Accumulated probability of survival (N=63)	Accumulated probability of survival		Log Rank
		Male (N=25)	Female (N=38)	p
24	0.74	0.70	0.84	0.114
48	0.61	0.64	0.58	
120	0.42	0.50	0.38	
240	0.31	0.31	0.32	
480	0.25	-	0.26	

**Figure 2 -:**
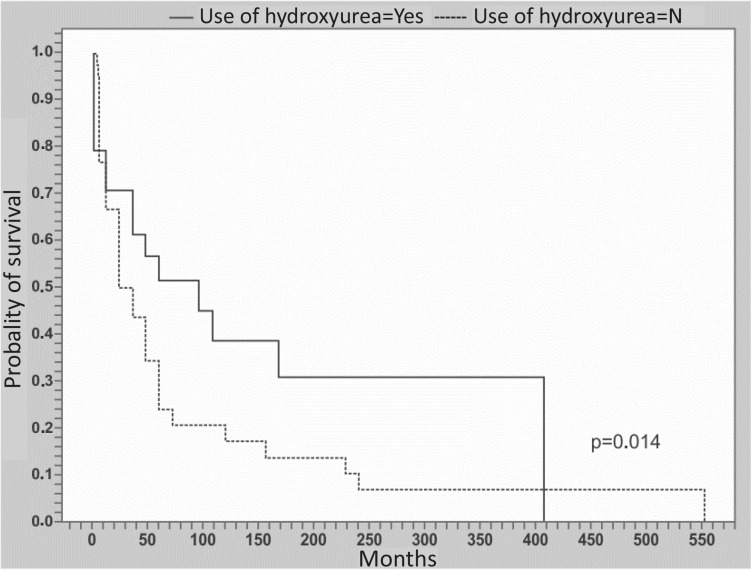
Comparative curve of estimated probability of survival, according to use or non-use of hydroxyurea, in two public hospitals of the state of Mato Grosso do Sul, Brazil, between 1980 and 2010 (n=63)

In analyzing the patients regarding their use or non-use of HU, the results showed that, at 24 months (two years), cumulative survival probability was 70% for the users of HU, compared with 50% of the non-users. At 48 months (four years), it was 62% for users of HU and 34% for non-users; at 120 months (10 years), it was 40% for users of HU, compared with 20% for the non-users; at 240 months (20 years), it was 32% for users of HU, compared with 8% of non-users; and at 480 months (40 years) there was 8% survival only in the case of patients not using the medication. Among the two groups, there was statistically significant difference (log rank=0.014) (Table 2). In Figure 2, one can note greater survival among the users of the medication.

**Table 2 -:** Accumulated probability of survival of patients with sickle cell disease; comparison between those who use hydroxyurea (N=39) and those who do not (N=24), in two public hospitals of the state of Mato Grosso do Sul, Brazil, between 1980 and 2010

Follow-up time (months)	Accumulated probability of survival with use of hydroxyurea (N=39)	Accumulated probability of survival without use of hydroxyurea (N=24)	Log Rank p
24	0.70	0.50	0.014
48	0.62	0.34	
120	0.40	0.20	
240	0.32	0.08	
480	-	0.08	

In comparing the time of use of HU between the sexes, it was observed that there was no statistically significant difference (*p*=0.285 - Mann-Whitney test); among women (n=25) the mean was 6.5±3.2 years, while among men (n=14) the mean was 5.3±3.1 years.

Of the ten deaths which occurred during the inpatient treatment, eight were female and 10 were male, in the ages varying between 17 and 42 years old, and with a mean age of 28.1 years old. Of these, eight had the Hb SS genotype (sickle cell anemia), and two, the Hb SC genotype (compound heterozygotes). 

In the comparison of the group of users of HU with non-users, it should be emphasized that, among the 10 cases of deaths, eight were women (five using HU) and two were male (one using HU), in the age range between 17 and 28 years old, with a mean age of 19.9 years old. The mean period of use of HU among the six deaths was 5.2 years. The majority (60%) of the 10 deaths occurred among patients who were not using HU, and in those who had been using it for less than five years. 

The causes of death were: Acute Respiratory Failure (ARF) (40%), Multiple Organ Failure (20%), Cardiogenic Shock (20%), Cerebrovascular Accident (CVA) (10%) and Septic Shock (10%).

## Discussion

This retrospective cohort, with observation of 30 years, evidences that in comparing the group of HU users with non-users of HU, the survival curve was greater among those who use the medication, for which the mean was six years of use, there being no association between time of use and survival between the sexes. These results corroborate the MSH study, which monitored individuals with sickle cell anemia over 17.5 years and observed that greater exposure to HU seems to have improved survival^(^ 12 ^)^. 

In the sample investigated, there is a predominance of adult females with Hb SS. These results are similar to those of the epidemiological study undertaken in Uberaba in the Brazilian state of Minas Gerais (MG) with 47 sickle cell patients, which reported a higher number of women (59.6%)^(^ 13 ^)^. No plausible reason was found for the predominance of females, as sickle cell disease is genetic and not related to sex. 

In relation to the mean age at the time the medication was indicated, in the second decade of life, and to the variation in the dosage of HU prescribed, an appropriate HU response was obtained, evidenced by the increase in the levels of fetal hemoglobin. This result demonstrates the effectiveness of this therapy, supported by the studies which sought to investigate the effects of HU in sickle cell anemia^(^ 10 ^-^ 14 ^)^.

The accumulated probability of global survival found in this study points to the lack of statistical significance in the comparison of male and female patients (Log Rank=0.114). However, the survival curve showed that in the first two years of life, the females presented a greater probability of survival than the males. In a cohort study undertaken in Jamaica, on the other hand, with 290 deaths between 3301 patients, a statistical difference was detected between the sexes, with greater survival for female patients (58.5 years) when compared with the males (53.0 years)^(^ 15 ^)^.

In the present study, the statistically significant difference attributed to the comparison of the group of users and non-users of HU indicates a survival curve which is greater for those who used the medication. The sample observed shows the exposure to HU over a mean of six years. These results evidence the benefits expected through its action, among which one finds a reduction in acute episodes, and the number of blood transfusions and episodes of inpatient treatment, resulting in greater survival and improvement of well-being and quality of life^(^ 6 ^)^. 

These findings are corroborated by a prospective study undertaken in Athens, Greece, with the objective of assessing the efficacy of HU, in which the mean period of follow-up was eight years for those using HU and five years for non-users. Results showed that HU produced a reduction in the frequency of severe pain crises, in transfusions, in episodes of inpatient treatment in hospital, and in the incidence of acute chest syndrome. The probability of 10 years of survival was 86% and 65% for patients using HU and not using HU, respectively^(^ 16 ^)^. 

One study with children and young adults, undertaken to assess the efficacy and toxicity of HU in the long term demonstrated that, in patients monitored for a minimum of five years, it was possible to ascertain a significant difference in the reduction of the number of episodes of hospitalization and the number of days of inpatient treatment involved over the period of treatment when compared with the period prior to the use of the therapy with HU. The probability of not suffering any event or vaso-occlusive crisis requiring hospitalization during the five years of treatment was 47% when compared with the period prior to the treatment (55%)^(^ 17 ^)^.

 In the comparison of the time of use of HU between the sexes in the present study, there was no statistical significance. This result is similar to that of a retrospective study undertaken in Georgia (United States of America), in which sex did not influence the survival of the 226 patients using HU^(^ 7 ^)^_._

In the present study, the results showed the recording of 87.3% of the patients with Hb SS; 12.7% with Hb SC; and deaths in the young age range. These findings confirm those described in the literature, emphasizing that sickle cell anemia (Hb SS), the homozygous state for hemoglobin S, represents the most common genotype, with the most serious clinical presentation of the disease^(^ 18 ^)^_._ In this case, it is appropriate to emphasize a Dutch study which, in analyzing the causes of death among patients with sickle cell disease in the period 1985 - 2007, detected that, among 298 children, 189 (63%) were Hb SS^(^ 19 ^)^_._

The present study ascertained the occurrence of mortality in the second decade of life. These findings are similar to those of a study undertaken in Minas Gerais (N=151 patients) in the period 1998 - 2007, in which, in the 11 deaths, the mean age was 33.5 years old, suggesting that in Brazil, patients with sickle cell disease die early; and that, therefore, the existence of an older adult population of those with the disease is not to be expected^(^ 20 ^)^. In this regard, it is worth emphasizing that approximately 88.9% (56) were between five and 40 years old, with the exception of one patient aged 63 years old. 

In this investigation, however, attention is called to the absence of deaths in patients with SCD in the age range of five to 12 years old, which differs from the literature^(^ 5 ^,^ 21 ^)^_. _One possible limiting factor of the present study is the fact that it is retrospective, being based in medical records, thus hindering the identification of the recording of deaths in this age range due to the lack of computerization in previous decades. 

It should be emphasized that, since the implementation of the Neonatal Screening Program in the State of Mato Grosso do Sul^(^ 22 ^)^, there have not been records of deaths in children with SCD, which represents an increase in life expectancy. Another aspect to be emphasized relates to the early diagnosis, promoting the instituting of preventive treatment measures, affording positive results regarding morbidity and mortality, and a greater probability of survival for the children^(^ 23 ^)^.

Among the 10 deaths which occurred in this study, attention is drawn to the seven occurrences verified in the age range between 17 and 28 years old, with a mean age of 19.9 years old. These findings relate to the study which indicates the following as possible factors contributing to the low age of death: late diagnosis, lack of guidance for the family in the light of the first signs of complications, the preventive measures against infections, the little-efficacious medical attendance during the clinical complications, and the irregular provision of medications through a governmental program^(^ 24 ^)^.

In relation to the genotypes, eight deaths were recorded with Hb SS and two with Hb SC, with mean ages of 26.7 and 33.5 years old, respectively. These findings are supported by a cohort study of analysis of survival in patients with sickle cell disease, which observed that those with phenotype SC survived for longer in comparison with those of phenotype SS. The survival of patients with Hb SS was 42 to 48 years old, and, in patients with Hb SC, from 60 to 68 years old ^(^ 4 ^)^. 

Another aspect to be considered in this study is that the majority (60%) of the 10 deaths occurred among patients who were not using HU and among those who had been using it for less than five years. In one randomized study of the MSH, 87.1% of the 31 deaths occurred in patients in the categories "never exposed to HU" and "with less than five years of use"^(^ 12 ^)^_._

Regarding the causes of death, Acute Respiratory Failure was responsible for four of the 10 deaths, as a consequence of pneumonia. Multiple Organ Failure occurred in two, related to infection/sepsis; and septic shock was responsible for one, due to sepsis. It follows that infection was the principal complication for the deaths in this study, a result similar to that of other findings on mortality in sickle cell disease^(^ 20 ^-^ 21 ^)^_._

Among the 10 deaths recorded in this study, cardiogenic shock was the cause of two, due to Congestive Heart Failure (CHF); and CVA was responsible for one. 

These complications directly compromise the function of vital organs and their associated with risk to life, with a predominance of, or being limited to, one age range. CHF is a typical late manifestation, requiring a long devolution of the tissue injury to be manifested, while a CVA may be evidenced in a much younger age range^(^ 25 ^)^_._

In this study, one of the deaths related to Cardiogenic Shock occurred at the age of 17 years old, while the other, caused by a CVA, occurred at the age of 26 years old. These findings differ from the study on morbidity and mortality in sickle cell disease which, in analyzing the causes of death, recorded one case of death from Cardiogenic Shock at the age of 34 years old, and one CVA at six years of age^(^ 20 ^)^_._ These differences in results are confirmed by the literature, which mentioned that the mechanisms underlying age predominance are not always present^(^ 25 ^)^_._

In this study, there were limiting factors for the analysis of some variables, resulting from the observational character of the study, however, without prejudice to the objectives established. 

## Conclusion

This study demonstrated the effectiveness of the use of HU in a cohort with retrospective data collection, with a mean of six years' exposure to the medication. 

In the comparison of the group of users of and non-users, the survival curve is greater for those using the medication, there being no association between time of use and survival between the sexes. 

The comparison of the group of users and non-users of HU shows the occurrence of 10 cases of deaths, with eight among women (five using HU) and two among men (one using HU). Of the total of deaths, seven are Hb SS, in the age range between 17 and 28 years old. 

The most frequent cause of death was ARF, followed by Multiple Organ Failure and Cardiogenic Shock. 

This study is relevant in that it raises scientific evidence regarding the advances of therapy with HU in SCD, which should be incorporated by the nurse into her care practice. These actions may facilitate this clientele's access to the different levels of care, as well as to medications - specifically hydroxyurea - aiming for a reduction in mortality and an increase in the survival of the patient with sickle cell disease.
